# Indirect effects of domestic and wild herbivores on butterflies in an African savanna

**DOI:** 10.1002/ece3.744

**Published:** 2013-09-05

**Authors:** Marit L Wilkerson, Leslie M Roche, Truman P Young

**Affiliations:** Department of Plant Sciences, University of CaliforniaDavis, California

**Keywords:** *Cadaba farinosa*, cattle–wildlife interactions, *Colotis* spp., herbivore exclusion, pollinators, rangeland system, resource webs, ungulate herbivory

## Abstract

Indirect interactions driven by livestock and wild herbivores are increasingly recognized as important aspects of community dynamics in savannas and rangelands. Large ungulate herbivores can both directly and indirectly impact the reproductive structures of plants, which in turn can affect the pollinators of those plants. We examined how wild herbivores and cattle each indirectly affect the abundance of a common pollinator butterfly taxon, *Colotis* spp., at a set of long-term, large herbivore exclosure plots in a semiarid savanna in central Kenya. We also examined effects of herbivore exclusion on the main food plant of *Colotis* spp., which was also the most common flowering species in our plots: the shrub *Cadaba farinosa*. The study was conducted in four types of experimental plots: cattle-only, wildlife-only, cattle and wildlife (all large herbivores), and no large herbivores. Across all plots, *Colotis* spp. abundances were positively correlated with both *Cadaba* flower numbers (adult food resources) and total *Cadaba* canopy area (larval food resources). Structural equation modeling (SEM) revealed that floral resources drove the abundance of *Colotis* butterflies. Excluding browsing wildlife increased the abundances of both *Cadaba* flowers and *Colotis* butterflies. However, flower numbers and *Colotis* spp. abundances were greater in plots with cattle herbivory than in plots that excluded all large herbivores. Our results suggest that wild browsing herbivores can suppress pollinator species whereas well-managed cattle use may benefit important pollinators and the plants that depend on them. This study documents a novel set of ecological interactions that demonstrate how both conservation and livelihood goals can be met in a working landscape with abundant wildlife and livestock.

## Introduction

For decades, ecologists have studied the impacts of ungulate herbivory on plant species diversity and richness (e.g., Milchunas et al. 1988; Noymeir 1995; Rambo and Faeth 1999; Weisberg and Bugmann 2003; Manier and Hobbs 2007). Many studies also have examined the indirect effects of herbivory on competitive interactions between large ungulates, especially between livestock and wild herbivores (e.g., Madhusudan 2004; Young et al. 2005; Wegge et al. 2006; Yoshihara et al. 2008b; Odadi et al. 2011). Less commonly studied are other indirect effects of herbivore-driven web interactions in natural systems (Paine 2000; Rooney and Waller 2003; Weisberg and Bugmann 2003; Pringle et al. 2007; Huffman et al. 2009; Peco et al. 2011). This study focuses on the indirect effects that large ungulate herbivores, domestic and wild, have on a nonplant taxa (butterflies) via a novel interaction. These indirect effects may have substantial impacts on native ecological communities.

Differential impacts of livestock and native wildlife on ecosystem structure and function have been largely unaddressed, with the exception of work on indirect competition among these herbivore guilds through food resources (Damhoureyeh and Hartnett 1997; Young et al. 2005; Manier and Hobbs 2007). However, it is well known that livestock and even similar wild herbivores differ in their foraging methods and preferences (Manier and Hobbs 2007; Odadi et al. 2007; Veblen and Young 2010). For example, Damhoureyeh and Hartnett (1997) found that bison and cattle differ significantly in their effects on native forb growth and reproduction. In addition, the dynamics between wild and domestic ungulate grazing can differ between wet and dry years (Odadi et al. 2009, 2011). As grazing can directly impact the structure, reproduction, and overall fitness of many plant species, knowing which type of grazer (wild vs. domestic) impacts different plant species can be critical to management and conservation decision-making.

One observed effect of herbivory is reduced allocation to reproductive structures in plants (Koptur et al. 1996; Niesenbaum 1996; Augustine and Frelich 1998; Hamback 2001; Goheen et al. 2007; Young and Augustine 2007). When plants are stressed by herbivory, they will (a) have fewer resources (photosynthate) to allocate and (b) reallocate resources to defense or regrowth rather than to reproductive structures (Whigham 1990; Cote et al. 2004). In addition, some herbivores eat floral structures (McCall and Irwin 2006). A few studies show that increased herbivory also indirectly reduces pollinator visits to flowering plants through a reduction in flower abundances or even through changes in floral morphology and other characters (Strauss 1997; Hamback 2001; Vazquez and Simberloff 2004). However, these studies focus on insect herbivory or artificially mimicked large mammal herbivory by clipping. There have been several descriptive (not controlled) studies suggesting that different domestic herbivores differently affect pollinator abundance or richness (Warren 1993; Carvell 2002; Öckinger et al. 2006; Yoshihara et al. 2008a), but controlled experimental studies of the effects of large herbivores on insects via their larval (leaf) or reproductive (pollen, nectar) resources are virtually nonexistent. In addition, the previous descriptive studies all focus on domestic herbivores. For working landscapes – which provide for ideally synergistic livelihood needs and conservation goals – research on both wildlife and domestic herbivore effects on pollinator species is also needed.

Here, we examine for the first time how wild herbivores and cattle indirectly affect the abundance of the most common butterfly taxon, *Colotis* spp., at a set of replicated long-term exclosure plots in an *Acacia* savanna rangeland system in central Kenya. The Kenya Long-term Exclosure Experiment (KLEE) has been the source of some of the few studies examining herbivore-driven indirect interactions on a variety of taxa, including small mammals, birds, invertebrate herbivores, spiders, fleas, snakes, lizards, and ants (Keesing 1998; Warui et al. 2005; McCauley et al. 2006; Pringle et al. 2007; Ogada et al. 2008; Palmer et al. 2008). Several of the KLEE studies demonstrate that cattle, wild megaherbivores (elephants and giraffes), and other wild ungulates have strongly different effects on the ecosystem they coinhabit (Warui et al. 2005; Riginos and Young 2007; Ogada et al. 2008; Riginos and Grace 2008; Veblen and Young 2010; Riginos et al. 2012). One of the unstudied aspects of these ecosystem-level studies is the interaction among domestic and wild herbivores, flowering plants, and pollinators.

For this study of indirect interactions, we hypothesized that: (1) herbivory treatment plots that have greater foliar abundance and/or flowering of a key shrub species will be correlated with greater butterfly abundances; (2) experimentally reduced levels of herbivory will result in increased foliar abundance and/or flowering and that this will be strongest in areas excluding both cattle and wildlife; and (3) due to differences in diet, wildlife and cattle will differ in their impacts on the plant species and, indirectly, on the butterflies. In particular, the wildlife guild, which includes browsers, will potentially have greater indirect effects on butterfly numbers through direct removal of shrub leaves and flowers; however, cattle, which graze grasses that compete with these shrubs, may actually increase butterfly abundance. To more closely examine the potential causal links between wildlife and domestic herbivores and *Colotis* spp., we utilized Bayesian structural equation modeling (SEM) to examine potential mechanisms by which herbivores indirectly influence *Colotis spp*. habitat selection via direct impacts to resource abundance. SEM is an effective multivariate analytical technique for addressing interactions in such complex natural systems (e.g., Anderson et al. 2007; Riginos and Young 2007; Roche et al. 2012). In the SEM analysis, we asked: What is the relative importance of adult resources (*Cadaba farinosa* flowers) and larval resources (*C. farinosa* leaf canopy) in driving *Colotis* spp. habitat selection.

Our results show strong links between shrub and flower densities and butterfly abundances and that, indeed, wildlife and cattle differ in their impacts on butterflies via the shrub species. In fact, we find that having cattle as the sole large herbivore species in this system is the most synergistic management treatment for the conservation of *C. farinosa* and *Colotis* spp.

## Material and Methods

### Study site

**Figure 1:**
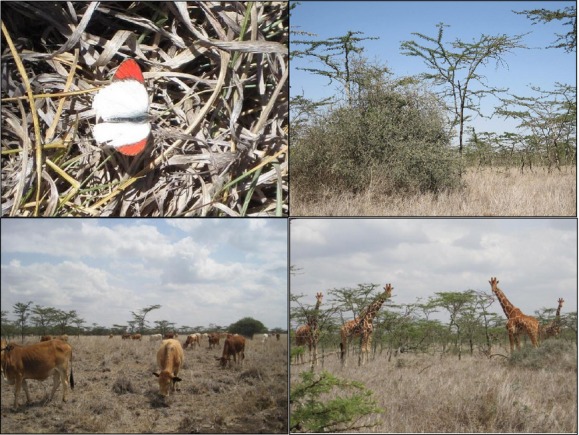
Photographs of focal study organisms (from upper left clockwise): example of *Colotis* spp. butterfly (Scarlet Tip butterfly, *Colotis danae eupompe*); *Cadaba farinosa* shrub in Acacia savanna system; example of common wildlife herbivore (giraffes, *Giraffa camelopardalis*); and common domestic herbivore (Boran cattle, *Bos indicus*).

This study was carried out in June–August 2007 and July–August 2009 in a set of herbivore exclosures set up in 1995 on Mpala Research Centre, a wildlife conservancy and working cattle ranch, in the Laikipia District of central Kenya (Young et al. 1998). All exclosures are located on “black cotton” vertisol soils and receive an average of 500–600 mm of rainfall per year. The habitat is dominated by *Acacia drepanolobium* and five main perennial grass species (Young et al. 1998). *Cadaba farinosa* is one of the most common woody plants (Fig. 1). The common large ungulate herbivores at the study site include domestic Boran cattle (*Bos indicus*), zebras (mainly *Equus burchelli*), Grant's gazelles (*Gazella granti*), giraffes (*Giraffa camelopardalis*), elephants (*Loxodonta africana*), oryx (*Oryx gazelle beisa*), hartebeests (*Alcelaphus buselaphus*), elands (*Tragelaphus oryx*), and buffaloes (*Syncerus caffer*) (Fig. 1).

The KLEE consists of three blocks (North, Central, and South), each divided into six 4-ha plots (for further detail, see Young et al. 1998). Each treatment plot differentially excludes and includes a unique combination of cattle, megaherbivores (elephants and giraffes), and other large wildlife (>15 kg) using a series of semipermeable barriers specially designed to exclude different guilds of herbivores. We used four treatment plots: only cattle allowed (C), only megaherbivores and wildlife allowed (MW), all herbivores allowed (MWC), and no large herbivores allowed (O). Note that the treatment notation refers to herbivore guilds that are included within the plots. In this study, there was no differentiation made between megaherbivores and other large native ungulates; all are called “wildlife” hereafter.

### Study species

We focused our study on butterflies in the genus *Colotis* (Pieridae), which are by far the most abundant butterflies in the study site (over 80% of observed butterfly individuals during study duration) (Fig. 1). There are five species of *Colotis* in the study area (*Colotis celimene*, *C. danae*, *C. eucharis*, *C. antevippe*, and *C. evagore*) and six common species of other butterfly genera. The larvae of all *Colotis* species specialize on plants in the family Capparaceae (Larsen 1991), and in our study area, *Colotis* adults preferentially visit *C. farinosa* (M. L. Wilkerson and T. P. Young, pers. obs.; D. Martins, pers. comm.). *Cadaba farinosa* accounts for >85% of all individual plants in the Capparaceae occurring in the study area.

*Cadaba farinosa* (Capparaceae) is a shrub that grows on heavy clay soils throughout eastern and southern Africa (Fig. 1). At KLEE, this species flowers at the end of the rainy seasons and the beginning of the dry seasons (usually in June and then again in December). During our surveys in July–August 2007 and July–August 2009, *Cadaba* shrubs were in bloom within the study area.

### *Cadaba* density and size

We estimated the density and mean canopy area of the *Cadaba* shrubs in the 12 sampled KLEE plots. Each KLEE plot was divided into sixteen 50 × 50 m subplots demarcated by nine internal posts. In 2009, at each of the nine posts within each sampled plot, we counted every *Cadaba* individual within 20 m of the post to avoid overlapping with adjacent subplots. For each individual shrub, we measured the canopy volume (length, width, and height).

### *Cadaba* flower counts

In 2007 and 2009, at each of the nine posts within each sampled subplot, we located the three nearest *Cadaba* shrubs within a 20-m radius around the post. We laid a 0.25 m^2^ quadrat over the part of the shrub closest to the post and counted the number of flowers inside the quadrat, through the entirety of the shrub's volume. Of the 108 total subplots, 50% had fewer than three bushes in a subplot. In those cases, all shrubs within the 20-m radius were surveyed for flower counts.

### Butterfly surveys

Before assessing the abundance of butterflies, we conducted a general survey of butterfly species composition within the KLEE plots in 2007. We collected all butterfly species observed until our rate of finding a new species dropped to less than one during 2 h of sampling. Counts required four mornings of collection. The collected butterflies were identified to species using Larsen (1991). This survey allowed us to gauge the species richness within the plots and to quickly identify butterflies to genus in the field. At a distance, the different *Colotis* spp. are difficult to differentiate, but any *Colotis* spp. was easy to distinguish from non-*Colotis* spp.

Butterfly counts were conducted between 0800 and 1200 over a 3-week period starting in late June 2007 and then again between 0800 and 1200 over a 3-week period starting in early July 2009. At each of the nine subplot posts, we counted all butterflies seen in a 30-sec period while slowly rotating around the post. This was done three times, in a row, during one visit and the greatest number of butterflies seen was recorded. Butterflies were recorded as either *Colotis* spp. or non-*Colotis* spp. Through multiple observations, it was clear that there was no double counting of butterflies at our spatiotemporal survey scale.

Butterfly activity was strongly affected by transient cloud cover and wind. As soon as a cloud passed over the sun, butterflies dropped to the vegetation. Therefore, counts were only conducted when there was full sun, little wind, and few clouds. Counts during which clouds passed over the sun were not used in the analysis.

### Statistical analysis

*Cadaba* densities, canopy areas, and flower counts were averaged across the individual shrubs counted to give mean values for each of the nine subplots in a treatment. Across both years, the numbers of *Colotis* butterflies were significantly different among experimental blocks, with the North block (which had more *Cadaba* plants) having more than twice as many *Colotis* butterflies than the other two blocks (df = 2, *F* = 5.87, *P* = 0.004). Therefore, independent variables were hierarchically nested within block in analysis of variance (ANOVA) tests on the effects of herbivore treatments on all dependent variables (*Colotis* spp. and *Cadaba* variables); linear regressions between dependent variables were also nested hierarchically within block and treatment. Flower counts were log transformed to satisfy ANOVA assumptions.

**Figure 2:**
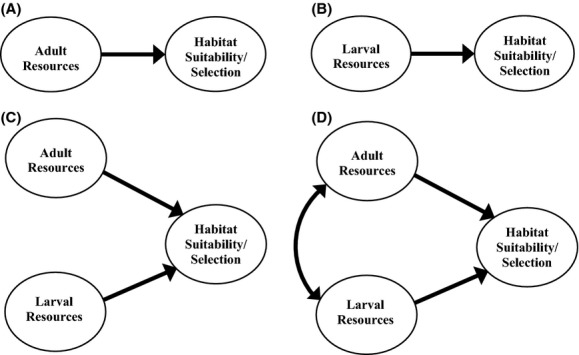
Conceptual model demonstrating the potential links between *Cadaba farinosa* larval and adult resources and habitat selection by a common pollinator butterfly genus, *Colotis*. The conceptual model includes the a priori hypothesized pathways of influence: (A) *Colotis* spp. habitat selection is driven by adult resources (pollen, nectar); (B) *Colotis* spp. habitat selection is driven by larval resources (shrub leaves); (C) both adult and larval resources have distinctive effects on *Colotis* spp. habitat selection; and (D) adult and larval resources have both direct and indirect effects (via environmental and/or genetic covariation between the two factors) on habitat selection. We evaluate this conceptual model via Bayesian structural equation model (SEM) analysis using a nested models approach.

To investigate the relative importance of *C. farinosa* larval and reproductive resources on butterfly habitat selection, we used two sets of models with the 2009 subplot level data from the wildlife (MW and MWC; “wildlife SEM”) and cattle (C; “cattle SEM”) treatments. For the wildlife SEM, MW, and MWC were combined because these treatments were not significantly different in terms of their effects on count of *Colotis* individuals or *Cadaba* variables (flower density, canopy cover, and bush density) in 2009. For each set of treatment data, we considered nested models that allowed us to examine the potential direct and indirect effects of *Cadaba* resources on *Colotis* habitat selection and suitability. *Cadaba* flower density was used as an indicator for adult resource abundance; total canopy and the number of *Cadaba* shrubs were indicators for larval resource abundance; and number of *Colotis* spp. individuals was an indicator for habitat suitability and selection. We first evaluated the individual direct effects of adult food resources (Fig. 2A) and larval food resources (Fig. 2B) as the main drivers of butterfly habitat selection. These resource factors may also directly affect habitat selection in distinctive ways; therefore, we examined whether the adult and larval food resources jointly contribute to butterfly habitat selection (Fig. 2C). Finally, adult and larval resource abundances are potentially correlated due to environmental and/or genetic covariation among traits (e.g., Gómez et al. 2009; Brock et al. 2010). Therefore, we also included a bivariate correlation between the adult and larval resource variables to account for potential indirect effects through covariance with the other resource factor (Fig. 2D). That is, in the full model (Fig. 2D), the total effect of each resource factor is partitioned into direct effects on butterfly habitat selection and individual indirect effects through covariance with the other resource factor. To account for nonindependence of subplots within treatment plots, random effects for plots were included in the models, and to account for higher level grouping in the wildlife SEMs, plot effects were nested within blocks (Pinheiro and Bates 2000; Gelman and Hill 2007; Rabe-Hesketh and Skrondal 2008).

Bayesian SEM analysis was performed using OpenBUGS software, which uses Markov chain Monte Carlo (MCMC) simulation based on Gibbs sampling algorithm to fit the models (Thomas et al. 2006). All indicators were log transformed to meet distributional assumptions and were standardized to aid model convergence (Congdon 2003). For all models, standardized regression coefficients were reported. Model convergence was assessed via trace plots with multiple chain sample values and a modified Gelman–Rubin statistic (Spiegelhalter et al. 2007). Model comparisons and goodness of fit were performed via the Deviance Information Criterion (DIC), a generalization of Akaike's Information Criterion (AIC) (Spiegelhalter et al. 2002). Reliability of individual model coefficients was examined via credible intervals (CI; the Bayesian equivalent of confidence intervals).

## Results

Both *Cadaba* canopy area and flower counts (log transformed) were correlated across 2007 and 2009 (*r*^2^ = 0.47, df = 1, *F* = 93.34, *P* < 0.0001 and *r*^2^ = 0.13, df = 1, *F* = 8.85, *P* = 0.004, area and counts, respectively) as were *Colotis* spp. butterfly counts across the 2 years (*r*^2^ = 0.23, df = 1, *F* = 31.35, *P* < 0.0001). To further examine year effects, we conducted an ANOVA on *Colotis* spp. counts, *Cadaba* canopy area, and *Cadaba* flowers (log transformed) using year, block, and treatment nested within block as the independent variables. The only significant year effect was for *Cadaba* flowers (df = 1, *P* = 0.002). There were significantly more *Cadaba* flowers in 2007 than in 2009 (LSQ mean of 1.79 and 1.22, respectively); this is likely due to the drought conditions across the Laikipia District in 2009. However, because there was only one variable that had a year difference and flower numbers were still correlated between years, we used 2-year averages for all dependent variables in the results below unless specifically stated otherwise.

### Univariate relationships between butterflies and flowering shrub variables

**Figure 3:**
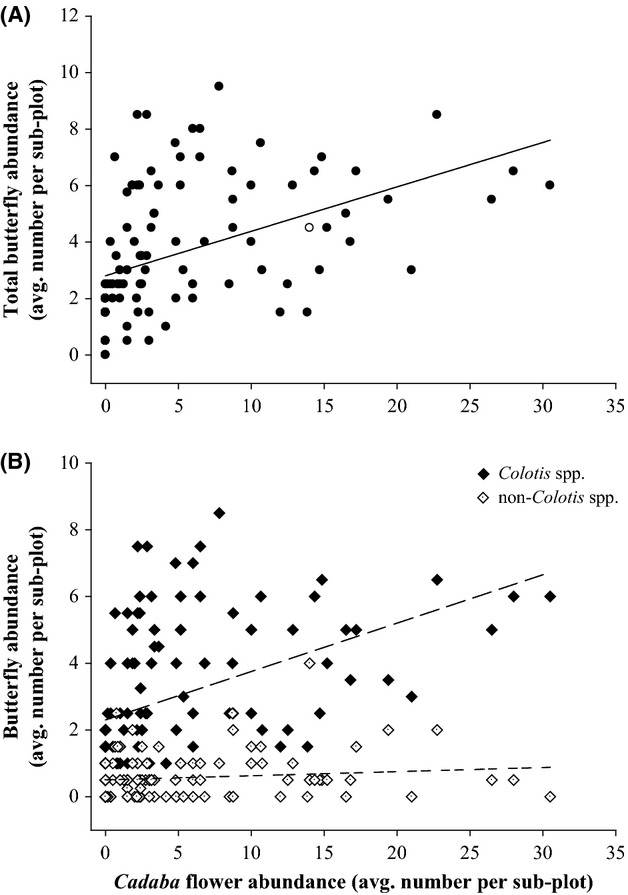
Regressions of butterfly abundances on *Cadaba farinosa* flowers. (A) There was a strong correlation across plots of all four herbivore treatments between *Cadaba* flowers (log transformed) and total butterflies per subplot (*R*^2^ = 0.203, ANOVA *P* = 0.02). (B) This relationship was driven by the butterfly of the common genus, *Colotis* (*R*^2^ = 0.245, ANOVA *P* = 0.02, long-dashed line) and not the other butterfly genera (*R*^2^ = 0.032, ANOVA *P* = 0.78, short-dashed line).

Over 80% of the butterflies counted in these surveys were in the genus *Colotis*. Averaging across years, there was a strong positive correlation across all plots, nested within block and treatment, between the number of *C. farinosa* flowers per quadrat (log transformed) and the number of total butterflies (*R*^2^ = 0.203, df = 3, *F* = 3.41, *P* = 0.02). This relationship was driven by the butterflies of the most common genus, *Colotis* (*R*^2^ = 0.245, df = 3, *F* = 3.68, *P* = 0.02), and not the other butterfly genera (*R*^2^ = 0.032, df = 3, *F* = 0.49, *P* = 0.78, Fig. 3).

Averaged across both years, there was also a strong correlation between *Cadaba* canopy area and flowers per quadrat (log transformed), nested within block (*R*^2^ = 0.321, df = 3, *F* = 11.21, *P* < 0.0001). Since we only had data on *Cadaba* density for 2009, we used only 2009 variables when determining correlations between density and other variables. In 2009, *Cadaba* canopy area and *Colotis* spp. numbers were both significantly positively correlated with *Cadaba* density, nested within block (*R*^2^ = 0.150, df = 3, *F* = 3.591, *P* = 0.04 and *R*^2^ = 0.340, df = 3, *F* = 10.49, *P* = 0.0004, respectively). *Cadaba* density was not significantly correlated with number of flowers.

### Indirect relationships between herbivores and butterflies

**Figure 4:**
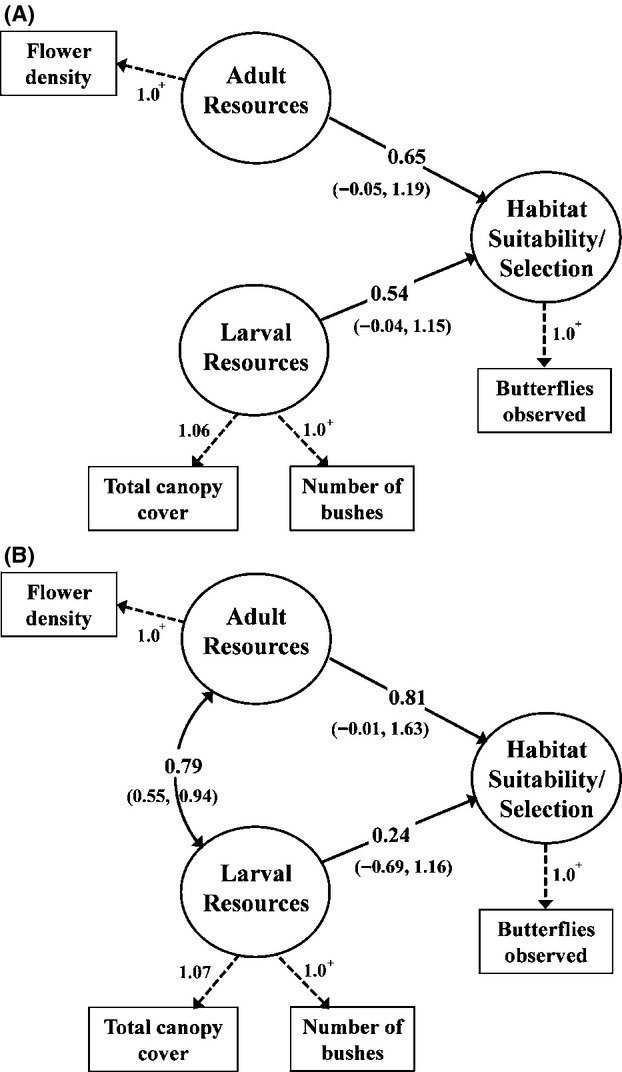
Wildlife SEM analysis demonstrated that *Colotis* spp. habitat suitability and selection mainly driven by adult resource abundance (A) Results of initial structural equation model linking independent latent variables of adult and larval resources to *Colotis* habitat selection for the wildlife treatments (MW and MWC). (B) Results for the Bayesian structural equation models after including the bivariate correlation between the adult and larval resource variables. Cattle SEMs (not shown) produced similar results. Latent variables are represented in ovals and measured variables (i.e., indicators) are represented in boxes. Dashed arrows represent the measurement models (relationships among the measured and latent variables) and the solid arrows represent the process model (structural relationships among the latent variables). Arrow values are the standardized regression coefficients; values in parentheses are the 90% credible intervals; +, fixed values.

For both the wildlife and cattle treatment data, Bayesian SEM analyses revealed that *Colotis* spp. habitat suitability and selection were driven by the abundance of the adult resource (*Cadaba* flowers), and not by the larval resource abundance (*Cadaba* canopy cover and density). The initial SEMs with uncorrelated latent (or unobserved) variables for adult and larval resources suggested that habitat use by *Colotis* spp. responded to both types of resources (Fig. 4A, cattle SEM not shown). However, after including a bivariate correlation (wildlife SEM: *r* = 0.79, 90% CI, 0.55−0.94; cattle SEM: *r* = 0.78, 90% CI, 0.44−0.95) between the adult and larval resource variables, the direct effect of larval resources was clearly no longer significant (wildlife SEM: standardized regression coef. = 0.24, 90% CI, −0.69 to 1.16; cattle SEM: standardized regression coef. = 0.70, 90% CI, −0.51 to 1.75; Fig. 4B, cattle SEM not shown). For both the wildlife and cattle analyses, the model with the lowest DIC included only the relationship between adult resources and *Colotis* spp. habitat selection (wildlife SEM: standardized regression coef. = 1.02, 90% CI, 0.67−1.34; cattle SEM: standardized regression coef. = 0.92, 90% CI, 0.69−1.12). Residual plots and DIC indicators showed reasonable model fits. The relative importance of total canopy cover and number of *Cadaba* shrubs as indicators of larval resources were comparable for both the wildlife and cattle SEMs (wildlife SEM: 1.07 vs. 1.0; cattle SEM: 0.9 vs. 1.0).

### Experimental manipulations

#### Wildlife versus no-wildlife effect on flowers and butterflies

Compared with plots without wildlife (C and O), *Cadaba* plants to which wildlife had access (MWC and MW) had 62% fewer flowers per plot (df = 5, *F* = 3.86, *P* = 0.004) and 28% fewer *Colotis* butterflies per plot (df = 5, *F* = 4.33, *P* = 0.002). Because of the strong correlation between *Cadaba* canopy area and flowers (see above) and the results from our SEM analysis that show the higher importance of adult resources (flowers) over larval resources (canopy and number of shrubs) (Fig. 4), we do not include canopy cover results here or below.

#### Cattle versus no-cattle effect on flowers and butterflies

The plots that had cattle (C, MWC) had 32% more *Colotis* butterflies than the plots without cattle (MW, O) (df = 5, *F* = 4.55, *P* = 0.001). There was no significant effect of cattle presence on *Cadaba* flowers.

#### Wildlife versus cattle effect on flowers and butterflies

**Figure 5:**
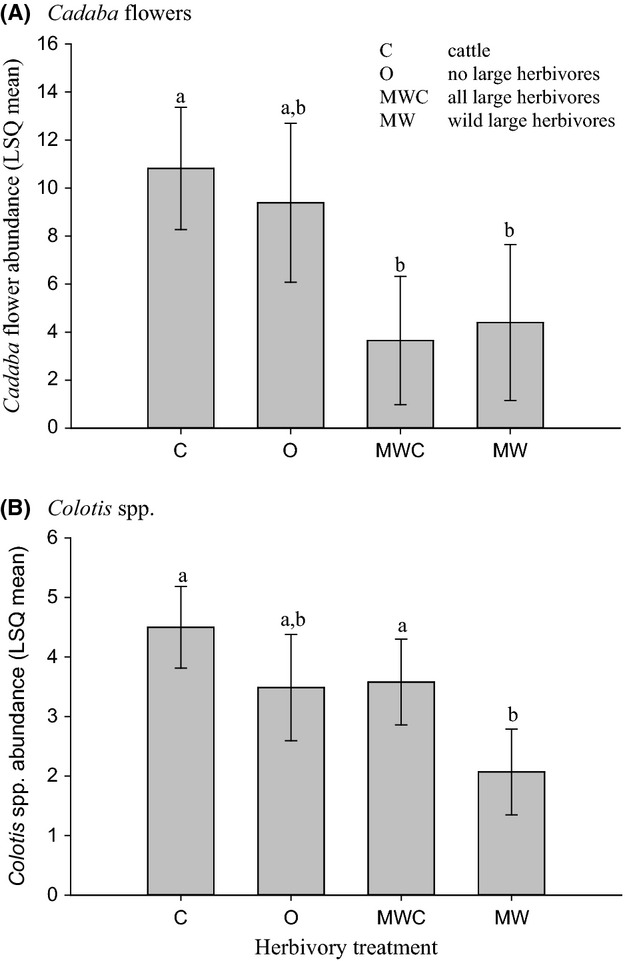
Averaged across year, both (A) *Cadaba* flower abundance, log transformed (note that raw flower counts are shown here for visibility ease, least square means, LSQ) and (B) *Colotis* spp. abundance (LSQ means) differed significantly among the four herbivory treatment types (ANOVA *P* = 0.0008 and *P* = 0.0001, respectively). For both dependent variables, the values in the cattle-only herbivory (C) differed significantly from those wildlife-only herbivory (MW); abundances of flowers and butterflies were highest in the C plots and lowest in MW plots. Bars sharing a letter were not statistically different (Tukey HSD test); error bars are two SEs from the mean.

Treatment types (C, MWC, MW, and O), nested within block, had significant effects on *Cadaba* flower numbers (df = 11, *F* = 2.61, *P* = 0.008) and *Colotis* spp. abundances (df = 11, *F* = 3.44, *P* = 0.001). When examining the simple effects among the four types of treatments, *Cadaba* and *Colotis* spp. values in treatments C (only cattle) were consistently significantly greater than in MW (only wildlife), whereas values in MWC (all herbivores) and O (no herbivores) treatments were intermediate (Fig. 5). Plots to which only cattle had access (C) had 64% more *Cadaba* flowers and 54% more *Colotis* spp. individuals than plots to which only wildlife had access (MW) (Fig. 5).

## Discussion

In our study area, *Colotis* butterflies used *Cadaba* spp. (and other Capparaceae) both as a larval food source (leaves) and as an adult food source (flower nectar). Individuals of *C. farinosa* were virtually the only Capparaceae present, and sites with more *Cadaba* individuals tended to have greater canopy area (more oviposition sites), more flowers, and more *Colotis* butterflies. Were these butterflies tracking adult resources (flowers) or larval resources (oviposition sites)? Our SEM analysis (Fig. 4) strongly suggested the former: for both cattle and wildlife treatment data, after controlling for the correlation between leaf canopy area and flower density, only adult resources significantly influenced *Colotis* butterfly abundances. Although larval resources and butterfly habitat selection are positively related, our SEM analysis shows that this apparent direct relationship is apparently due to a positive covariance between larval and adult resources (e.g., due to environmental and/or genetic covariation among traits) – and the adult resources are likely the underlying driver of *Colotis* habitat selection.

Both domestic and wild herbivores had strong indirect effects on this common genus of floral visitors. *Cadaba* plants had more flowers in plots where cattle were present and wildlife were excluded, and this had an indirect effect on the abundance of *Colotis* spp. This indirect effect supports our first hypothesis that *Cadaba* flowers and *Colotis* spp. abundances would be positively correlated. The higher flower counts in plots without wildlife is logical because wildlife browse on *Cadaba* shrubs and cattle largely do not (Odadi 2003). The pattern of wildlife browsing on woody vegetation and cattle grazing primarily on herbaceous species is well corroborated in this system and others (Wegge et al. 2006; Yoshihara et al. 2008b; Augustine et al. 2009). These results also support our third hypothesis that wildlife and cattle have different effects on *Cadaba* and butterflies. In the absence of wild herbivores, the presence of cattle appeared to be linked with an increase in the abundance of *Colotis* spp. as compared to the total exclusion plots. The absence of all large wildlife herbivory did not lead to an increase in *Cadaba* flowers and their floral visitors; in fact, it did the opposite. Similarly, surveys of nonexperimental sites differing in grazing by domestic herbivores have shown that some species (often browsers) reduce pollinator abundance or richness, whereas others (grazers) may increase it (Carvell 2002; Öckinger et al. 2006; Yoshihara et al. 2008a).

The mechanistic reasons why total herbivore exclusion did not result in the highest foliar or floral abundance of all the treatments in our study remain unclear. Other studies have shown that both extremes of herbivory (i.e., either the complete absence of herbivory or very intense grazing) often produce negative effects on forbs and shrubs (Smart et al. 1985; Milchunas et al. 1988; Cote et al. 2004). Sjodin et al. (2008) found decreased butterfly abundances in long-ungrazed pastures, compared to pastures grazed by cattle or horses. During the past decade in the KLEE plots, total exclusion initially led to dense rank grass followed by increased mortality of several herbaceous species and then to increases in certain herbaceous forbs, such as *Helichrysum glumaceum* (T. P. Young, unpubl. data). Changes in the herbaceous community in these large-herbivore total exclusion plots may have led to increased competitive pressure on *Cadaba* or altered some other aspect of a disturbance system that large herbivores provide. Several studies have explored the different pathways by which herbivore presence impacts herbaceous communities, focusing often on the competitive release hypothesis or habitat modification disturbances (Hartnett et al. 1996; Jutila and Grace 2002; Rooney and Waller 2003). There are many possible ways in which large herbivores can impact the herbaceous community or even a single plant species.

Whatever the pathway, having large herbivores has been shown to be beneficial to both grazed and ungrazed plant species in many systems. In a tallgrass prairie system, Fahnestock and Knapp (1994) found that bison herbivory on grasses indirectly facilitates forb growth through increased light availability and reduced competition. Other studies support the idea that grazing by large native and/or domestic ungulates increases floristic biodiversity relative to ungrazed areas (e.g., Collins et al. 1998; Hickman et al. 2004; Manier and Hobbs 2007). Our study's findings also support the beneficial role of large herbivores (in this case, cattle) on a common shrub species.

In addition to affecting the flowering of key plants, a lack of large herbivores might affect butterflies in other ways. High levels of elephant disturbance in Tanzania increase butterfly abundance and diversity (Bonnington et al. 2008). The authors conclude that elephant disturbance increases habitat heterogeneity (largely caused by feeding and moving behavior) and changes floral dynamics by increasing the numbers of plants used by butterflies. Their finding regarding the beneficial influence of herbivore-mediated disturbance parallels our result that cattle, which do not eat our focal plant species, were beneficial to *Colotis* butterflies. Butterflies often do better with a low or moderate level of disturbance in their habitat compared to no disturbance (Hamer et al. 2003; Bonnington et al. 2008).

Cattle herbivory often negatively impacts many native plant species, altering community composition and structure (Vazquez and Simberloff 2004; Young et al. 2005; Young and Augustine 2007). This current study, however, suggests that the complete exclusion of all herbivores may have deleterious effects in a system with a long evolutionary history of herbivory, and that in the absence of wildlife, the presence of domesticated livestock at moderate stocking densities may actually enhance the fitness and diversity of different taxa. This positive aspect of livestock is potentially important in an era of great reductions in wildlife numbers throughout Africa. In Kenya and other East African countries, livestock management and plant and wildlife conservation are often in opposition (Kinyua et al. 2000; Lamprey and Reid 2004; Gadd 2005; Georgiadis et al. 2007a). However, much research has demonstrated that well-managed cattle at moderate stocking densities can coexist successfully with wild herbivores, both playing roles in promoting the biodiversity and health of a system (Young et al. 1998; Georgiadis et al. 2007b; Augustine et al. 2009; Riginos et al. 2012).

These results represent a novel interaction in which herbivores indirectly affect butterflies primarily via their effects on their adult food plants. The butterfly response documented here demonstrates differences in habitat use, which is likely driven by butterfly movement into plots with more *Cadaba* flowers. We have no direct evidence that this would be paralleled by a change in overall *Colotis* spp. population size across an entire landscape. However, we believe that a large-scale effect would be a reasonable response to posit given the interactions described above. In any case, the data reported here represent some of the only experimental evidence that large mammal herbivory affects invertebrate floral visitors. Acknowledging the presence of and understanding the mechanisms behind the indirect effects of large herbivores on multiple taxa is crucial, especially as pastoral and agricultural activities increase, resulting in an increase in the frequency of interactions between native wildlife and domestic herbivores on working landscapes. Future research that investigates the exact mechanisms behind the observed decrease of *Cadaba* flowers and *Colotis* butterflies by wildlife herbivory and the apparent opposite effect by cattle presence will increase our understanding of these complex interactions. This and future studies will further basic scientific understanding of food and other resource webs and aid conservation efforts by elucidating how domestic and native biodiversity can best share the same ecosystems.
